# Pulmonary valve endocarditis in adults with congenital heart disease: the role of echocardiography in a case series

**DOI:** 10.1093/ehjcr/ytaa195

**Published:** 2020-09-19

**Authors:** Flavia Fusco, Giancarlo Scognamiglio, Anna Correra, Assunta Merola, Diego Colonna, Michela Palma, Emanuele Romeo, Berardo Sarubbi

**Affiliations:** Adult Congenital Heart Disease Unit, Department of Cardiology, Monaldi Hospital, Leonardo Bianchi Street 1, 80131 Naples, Italy

**Keywords:** Endocarditis, Pulmonary valve, Congenital heart disease, Echocardiography, Pulmonary prosthesis, Case series

## Abstract

**Background:**

Pulmonary valve (PV) endocarditis is a frequent complication during follow-up in patients with repaired right ventricular outflow tract (RVOT) obstruction and poses relevant diagnostic and treatment challenges. We aimed to describe in details the possible different clinical presentations of this rare condition and to highlight the role of both transthoracic and transoesophageal echocardiography which, in experienced hands, may provide comprehensive useful information for the clinicians.

**Case summary:**

We below describe the clinical presentation and the echo findings of three cases of pulmonary valve endocarditis complicating disease course after different repair modalities of congenital right ventricular outflow tract obstruction.

**Discussion:**

The present case series outlines the diagnostic challenges of this increasingly frequent complication during follow-up of patients with congenital RVOT dysfunction after both surgical and percutaneous repair. Despite the diffusion of multimodality imaging, echocardiography with PV-dedicated views play a pivotal role in diagnosing such condition and guiding clinical management. Furthermore, this case series highlight that the suspicion of infective endocarditis should be raised whenever a sudden increase in transvalvular gradient is found during follow-up.

**Learning points**

- In adults with congenital heart disease, infective endocarditis (IE) may involve both native and prosthetic pulmonary valve (PV) after surgical or transcatheter repair.
- In the era of multimodality imaging, transthoracic and transoesophageal echocardiography with PV-dedicated views play a pivotal role in diagnosing such condition and guiding clinical management.
- The predominant echo feature of PV endocarditis is right ventricular outflow tract obstruction. Infective endocarditis should be suspected whenever a sudden increase in transvalvular gradient is found during follow-up.

## Introduction

In the general population, infective endocarditis (IE) involving pulmonary valve (PV) is an extremely rare event, accounting for 1.5–2% of all cases.^1^ However, the risk of PV endocarditis significantly rises in the rapidly growing subset of adults with repaired congenital right ventricular outflow tract (RVOT) defects.^2^^,^^3^

Here, we present three cases of PV endocarditis complicating three different repair modalities of congenital RVOT obstruction.

## Timeline

**Table:** 

	Case 1	Case 2	Case 3
Cardiac diagnosis	Double outlet right ventricle with pulmonary stenosis (PS)	Congenitally corrected transposition of the great arteries with pulmonary atresia, ventricular septal defect (VSD), and major aortopulmonary collateral arteries	Tetralogy of Fallot (TOF)
Surgical history	Blalock–Taussig (BT) shunt during infancy	BT shunt and transcatheter pulmonary valve (PV) perforation during infancy	TOF repair during infancy
	Right ventricle (RV) to pulmonary artery (PA) conduit at the age of 12	PV balloon dilatation at the age of 19	RV to PA contegra conduit at the age of 14
	PPVI at the age of 21		
Age at presentation	24 years old	19 years old	27 years old
Time from last PV procedure	3 years	1 month	13 years
Clinical presentation	Fever following a dental abscess	Persisting fever after cardiac procedure	Exertional syncope
	Raised inflammatory markers	Raised inflammatory markers	Intermittent fever with GI symptoms 1 month earlier
Echo findings	Mobile filamentous echogenic mass	Large mobile vegetation PV overriding unrestricted VSD	Degenerated and thickened PV leaflets with multiple mobile masses
Localization of vegetation	Attached to the stent in right ventricular outflow tract	Ventricular side of the PV	Ventricular side of the PV
Best echo views	High parasternal view	Subcostal view	Transoesophageal echocardiogram with upper oesophageal views
			Conduit is not well seen on transthoracic echocardiogram
PV function	Normal function	Severe PS	Severe PS and moderate pulmonary regurgitation
Blood cultures	*Streptococcus anginosus*	*Streptococcus sanguinis*	*Staphylococcus aureus*
Treatment	Medical management with 6 weeks of antibiotics	Medical management with 6 weeks of antibiotics	Urgent surgical treatment
Follow-up (FU)	At 2-month FU: vegetation reduced in size, less mobile	At 6-month of FU: complete resolution of vegetation	Surgical PV replacement with a homograft
	Conservative management	Conservative management	Good function of the PV at 3-month FU
	At 6-month FU: complete resolution of vegetation		

## Case presentation

### Case 1

**Figure 1:**
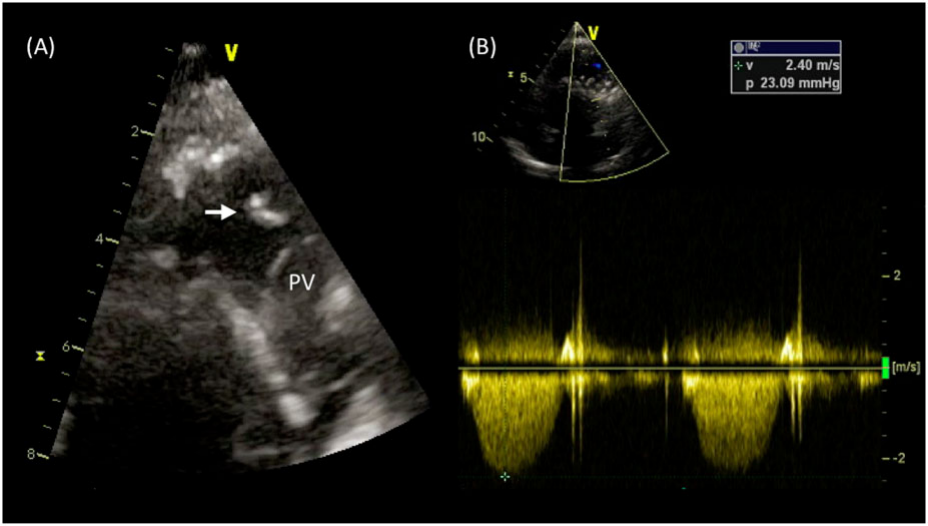
A case of pulmonary valve endocarditis in a patient with double outlet right ventricle and pulmonary stenosis after percutaneous pulmonary valve implant (Case 1). (*A*) High parasternal echo view: a mobile filamentous echogenic mass attached to the struts of the proximal segment of the stent in right ventricular outflow tract is visualized. (*B*) Continuous-wave Doppler signal demonstrating normally functioning pulmonary valve. Arrow, vegetation; PV, pulmonary valve; RVOT, right ventricular outflow tract.

**Figure 2:**
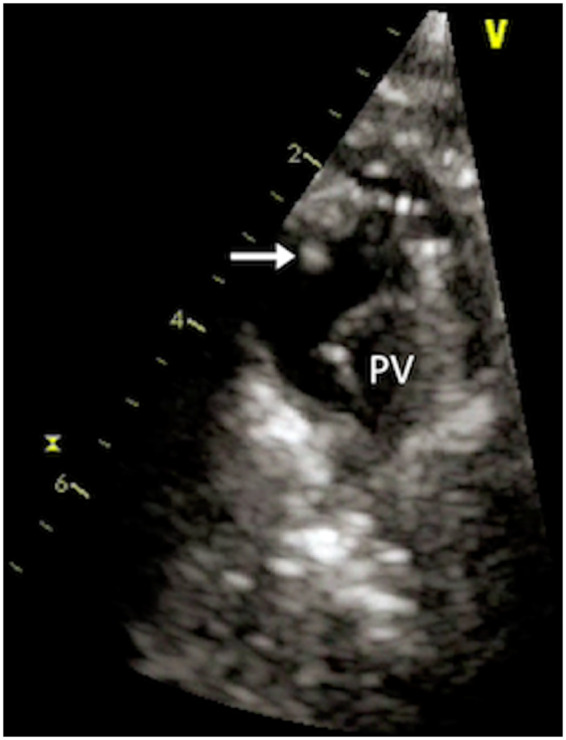
Same patient of *Figure 1* after 6 weeks of antibiotic treatment (Case 1). Transthoracic echo showed significant vegetation size reduction. Arrow, vegetation; PV, pulmonary valve.

A 24-year-old man with double outlet right ventricle and pulmonary stenosis (PS), with previous surgical palliation with Blalock–Taussig (BT) shunt in early infancy and subsequent biventricular repair with right ventricle (RV) to pulmonary artery (PA) conduit placement, developed a progressive conduit stenosis during follow-up. A percutaneous pulmonary prosthesis implant with a Melody^TM^ valve (Medtronic, MN, USA) was successfully performed with symptoms relief. Three years later, the patient was referred to our Adult Congenital Heart Disease (ACHD) Unit for fever following a dental abscess and persisting despite appropriate antibiotic treatment. On physical examination at admission, the patient was febrile, the heart rate (HR) was 110 b.p.m., blood pressure was 100/60 mmHg, and the oxygen saturation was 99% on room air. Inflammatory markers were markedly raised and transthoracic echocardiogram (TTE) showed normal biventricular systolic function [left ventricular ejection fraction (LVEF) 60%, tricuspid annular plane systolic excursion (TAPSE) 20 mm] and a well-seated and normally functioning bioprosthetic PV with thin and mobile leaflets. However, from high parasternal short-axis view, a mobile filamentous echogenic mass was visualized attached to the struts of the proximal segment of the stent in RVOT (*Figure 1A* and *B* and Supplementary material online, *Videos S^1^* and *S^2^*). No signs of pulmonary embolization were demonstrated at the positron emission tomography (PET)/computed tomography (CT) imaging. Serial blood cultures identified *Streptococcus anginosus* as the causative microorganism and, accordingly, a treatment with teicoplanine was commenced. Six weeks later, the patient was afebrile and in good clinical status and the inflammatory markers returned to normal values. However, TTE at 2-month follow-up showed persistent PV mass, albeit significantly reduced in size, hyperechogenic, and less mobile (*Figure 2* and Supplementary material online, *Video S^3^*). Considering that the patient was haemodynamically stable with no signs of active infection, and relying on the echocardiographic features suggestive of low risk of embolization, a conservative watchful management was followed. The 6-month follow-up TTE showed complete resolution of the vegetation.

### Case 2

**Figure 3:**
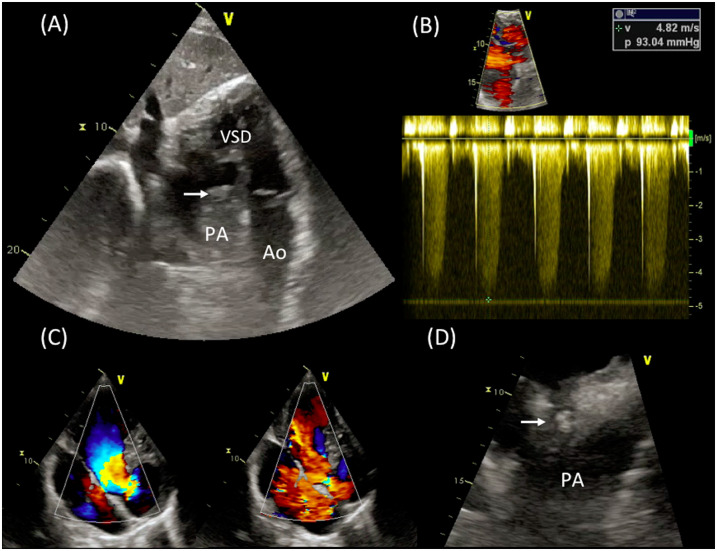
Pulmonary valve endocarditis in a patient with complex cardiac diagnosis including congenitally corrected transposition of the great arteries and pulmonary stenosis treated with balloon pulmonary valve dilatation (Case 2). (*A*) Subcostal view demonstrating parallel course of the great arteries with stenotic pulmonary valve overriding an unrestricted ventricular septal defect. An echogenic mobile vegetation attached to the ventricular side of the pulmonary valve is also seen (arrow). (*B*) Continuous wave Doppler signal showing pulmonary valve transvalvular gradient. (*C*) Bidirectional flow across the subpulmonary ventricular septal defect. (*D*) Zoomed view on the pulmonary valve mass from a modified apical view. Ao, aorta; Arrow, vegetation; PA, pulmonary artery; PV, pulmonary valve; VSD, ventricular septal defect.

**Figure 4:**
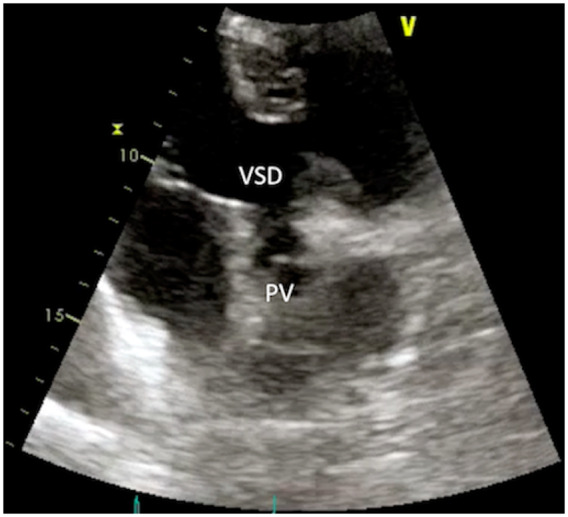
Same patient of *Figure 3* at 6-month follow-up (Case 2). Zoomed echo view from modified apical view showing complete resolution of the vegetation on the pulmonary valve. PV, pulmonary valve; VSD, ventricular septal defect.

A 19-year-old man with complex congenital heart disease was referred to our division for persisting fever 1 month after a percutaneous procedure. The cardiac diagnosis was mesocardia, congenitally corrected transposition of the great arteries with non-restrictive ventricular septal defect (VSD), pulmonary atresia with hypoplastic pulmonary arteries supplied by numerous major aortopulmonary collateral arteries. During infancy, he underwent bilateral BT shunts followed by transcatheter perforation of the PV. As significant PS with clinical signs of severe pulmonary hypoperfusion persisted, 1 month before attending our ACHD Unit, a percutaneous PV balloon dilatation was successfully performed at other institution with subsequent improvement in effort tolerance. On admission at our unit, the patient reported severe shortness of breath. On physical examination, the patient was febrile, HR was 105 b.p.m., room air oxygen saturation was 90%, and a harsh 4/6 Levine systolic murmur was appreciable at the cardiac base. At TTE, a narrow RVOT with a dysplastic PV overriding a large, unrestricted VSD were visualized from subcostal and modified apical views (*Figure 3A* and *C*). Normal biventricular systolic function (LVEF 58%, TAPSE 18 mm) was demonstrated. Doppler analysis revealed severe PS with peak gradient measured at 93 mmHg with no significant pulmonary regurgitation (PR) detectable (*Figure 3B*). Moreover, a large and mobile vegetation was visualized attached to the ventricular side of the PV (*Figure 3A*–*D* and Supplementary material online, *Videos S4* and *S5*). No signs of pulmonary embolization were demonstrated at the PET/CT imaging. *Streptococcus sanguinis* was isolated from blood cultures and adequate antibiotic regimen with gentamicin and ceftriaxone was administered for 6 weeks with clinical improvement and echo evidence of progressive reduction in size of the vegetation. At 6-month follow-up, TTE showed complete resolution of the PV vegetation (*Figure 4* and Supplementary material online, *Video S6*), and the patient was managed conservatively.

### Case 3

**Figure 5:**
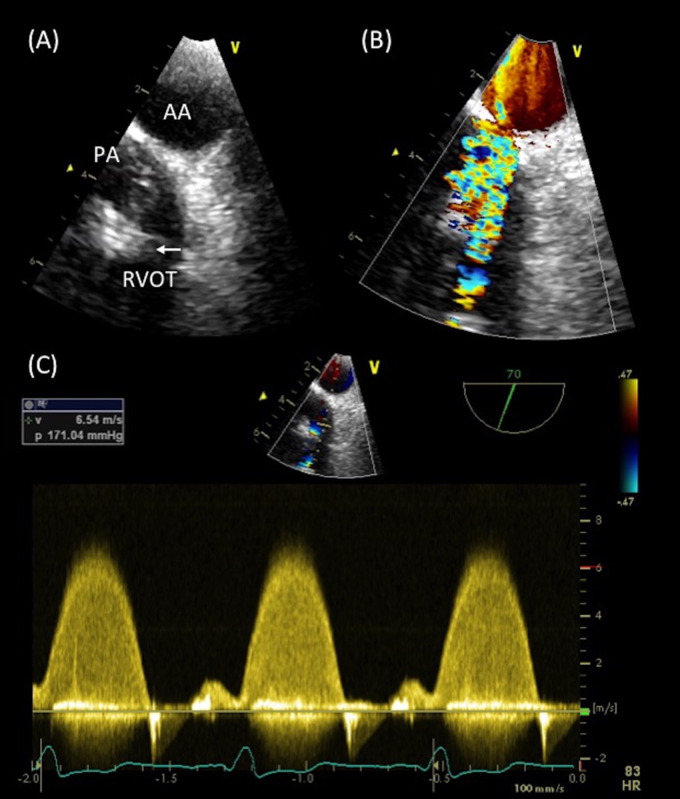
Transoesophageal echocardiogram images in a patient with repaired tetralogy of Fallot with a right ventricle to pulmonary artery conduit (Case 3). (*A*) Upper oesophageal transoesophageal echocardiogram views at 90° displaying degenerated and thickened pulmonary valve leaflets with numerous masses. (*B*) Colour Doppler images demonstrated significant flow acceleration across the valve. (*C*) Doppler signal proved severe pulmonary stenosis and moderate pulmonary regurgitation. AA, aortic arch; Arrow, vegetation; PA, pulmonary artery; PV, pulmonary valve; RV, right ventricle; RVOT, right ventricle outflow tract.

A 27-year-old man with tetralogy of Fallot (TOF) repaired during childhood and reoperated at the age of 14 with an RV to PA Contegra^®^ (Medtronic, MN, USA) conduit placement, presented to our ACHD Unit after a syncopal episode occurred during a mild effort. He reported, 1 month earlier, an episode of intermittent fever associated with gastrointestinal symptoms. On admission, he was afebrile and well perfused with normal vital signs. A harsh 4/6 Levine systolic and a 3/6 diastolic murmur were heard at the cardiac base. Routine blood tests demonstrated elevation of the inflammatory markers. TTE showed dilated and mildly hypokinetic RV [TAPSE 14 mm, fractional area change (FAC) 30%] with significantly raised RV systolic pressure. Although the conduit was non-visible on 2D echocardiography, Doppler signal at RVOT level revealed severe PS with peak gradient of 171 mmHg (*Figure 5C*). LV systolic function was preserved (LVEF 60%). The transoesophageal echocardiogram (TOE) with upper oesophageal (UE) views allowed clear visualization of the severely stenotic conduit with degenerated and thickened PV leaflets determining moderate PR. Moreover, multiple small, mobile masses arising from the ventricular side of the PV were visualized (*Figure 5A* and *B* and Supplementary material online, *Videos S7* and *S8*). The CT imaging demonstrated severely reduced inner conduit lumen size with a minimum diameter of 3 mm, while no signs of embolization were found. *Staphylococcus aureus* was isolated from serial blood cultures. Based on the TOE results, the patient underwent a successful surgical PV replacement with a homograft.

## Discussion

The findings from our series show that, in ACHD patients, PV IE may involve both native and bioprosthetic PV after surgical or transcatheter repair leading to relevant diagnostic and clinical difficulties during follow-up.

Nowadays, PV is the cardiac valve less frequently affected by IE, with the majority of cases confined to patients with congenital RVOT dysfunction. In the last decades, surgical advances allowed to a growing proportion of patients with successfully repaired congenital heart disease to reach adulthood with residual lesions and sequelae, often requiring further treatment during their lifetimes. Accordingly, the increase in prevalence of patients who had undergone either surgical or percutaneous PV procedures results in a parallel increase in the population at risk for IE. In fact, both surgical PV replacement^2^ and percutaneous procedures^3^ predispose to IE during follow-up. In particular, evidence from several reports in the literature suggested that bovine-derived PV prosthesis might be particularly susceptible to infections.^4^

Even though echocardiography remains the mainstay imaging modality in the diagnostic flowchart of IE, difficulties in images acquisition resulting from the variable position of the conduits or shadowing artefacts from stented valves may prevent accurate PV leaflets visualization. Notwithstanding, our case series demonstrates that, in tertiary centres with expertise in imaging in ACHD, echocardiography provides fundamental and reliable morphological and functional information for a correct diagnosis, for guiding the management strategy and for monitoring treatment response. Multiple and off-axis unconventional echo views may be helpful to clearly visualize all the structures in the RVOT.

Transoesophageal echocardiogram is often underused to image RVOT because PV visualization may be limited by its anterior position in chest, thus appearing in the far field. However, TOE role in suspected PV endocarditis should not be neglected, especially when TTE is insufficient to accurately rule out the involvement of other cardiac structures, such as other valves and subvalvular apparatus, patches or intra-cardiac shunts. Furthermore, using PV-dedicated views, TOE may in some cases allows a comprehensive visualization of the anteriorly seated valved conduits as shown in Case 3.

Moreover, it should be noted that, unlike IE involving other cardiac valves, PV endocarditis more often presents with a stenotic lesion, as previous studies have demonstrated.^5^ The high burden of infected material described in the surgical resection specimens, along with some degree of inflammatory thickening and thrombosis of the bioprosthetic leaflets have been hypothesized as possible causes.^6^ However, non-infective subclinical thrombosis has been demonstrated in explanted prosthetic PV^7^ and it could be speculated that thrombotic material deposition and consequent turbulent flow across the valve may predispose to infections. Even though further studies are necessary to clarify the physiopathologic substrate of PV endocarditis presenting with stenosis, this unique feature distinguishing PV endocarditis should raise the suspicion of valve infection whenever a sudden increase in transvalvular gradient is found during follow-up.

## Conclusions

Pulmonary valve endocarditis is a frequent complication during follow-up of patients with congenital RVOT dysfunction after both surgical and percutaneous repair. Echo images may be challenging, nevertheless, using unconventional PV-dedicated views, echocardiography provides fundamental info not just for diagnosis but also for treatment guidance.

## Lead author biography

**Figure:**
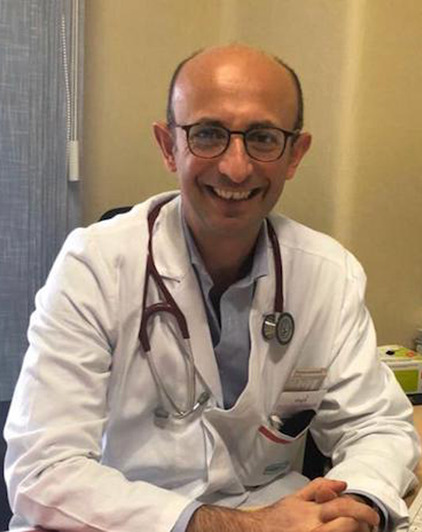


Dr Giancarlo Scognamiglio, MD, PhD, is a Consultant Cardiologist and Lead of the Imaging Programme in Adult Congenital Heart Disease at the GUCH Unit of Monaldi Hospital in Naples, Italy. During his training, he specialized in transthoracic and transoesophageal echocardiography and congenital heart disease. His fields of interest include congenital heart disease, valvular heart disease, pulmonary hypertension, ICU, transthoracic and transoesophageal echocardiography, and 3D echo. He is author of chapters in 4 scientific books and more than 40 scientific papers on Peer-Reviewed Journals. Dr Scognamiglio is Professor of Congenital Heart Disease in the Specialty Training Programme in Cardiology at the University of Campania Luigi Vanvitelli.

## Supplementary material

Supplementary material is available at *European Heart Journal - Case Reports* online.

**Slide sets:** A fully edited slide set detailing this case and suitable for local presentation is available online as Supplementary data.

**Consent:** The author/s confirm that written consent for submission and publication of this case report including image(s) and associated text has been obtained from the patients in line with COPE guidance.

**Conflict of interest:** none declared.

## Supplementary Material

ytaa195_Supplementary_Data
