# Supplementary material for: Pulmonary valve endocarditis in adults with congenital heart disease: the role of echocardiography in a case series
Source: Eur Heart J Case Rep. 2020 Sep 19;4(5):1–7. doi: 10.1093/ehjcr/ytaa195 (PMC7649497; doi:10.1093/ehjcr/ytaa195)
Supplement: ytaa195_Supplementary_Data [file ytaa195_supplementary_data.zip › ytaa195_Supplementary_Data/slide set2.pptx]

## Slide 1
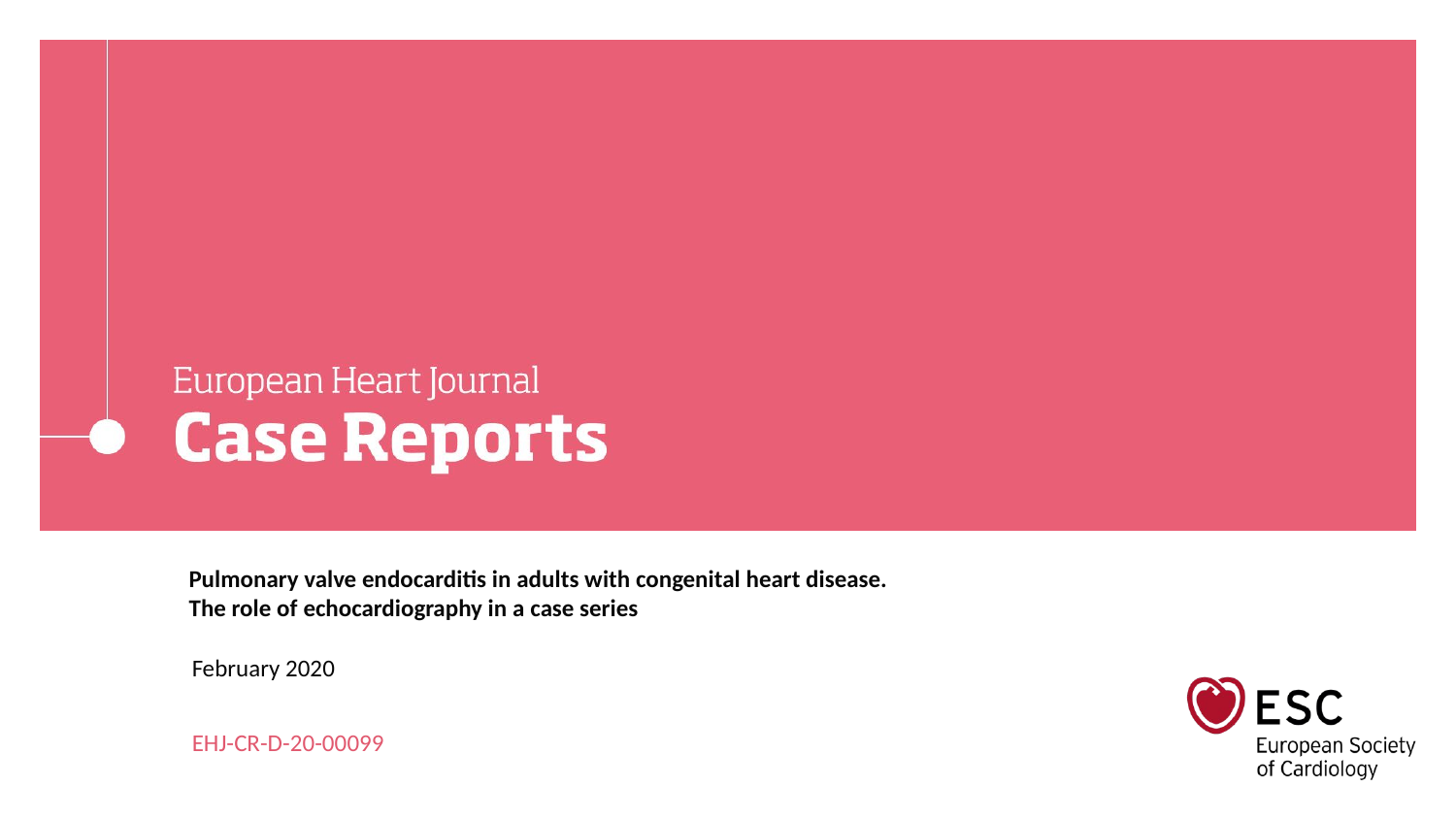

# Pulmonary valve endocarditis in adults with congenital heart disease. The role of echocardiography in a case series
February 2020
EHJ-CR-D-20-00099

## Slide 2
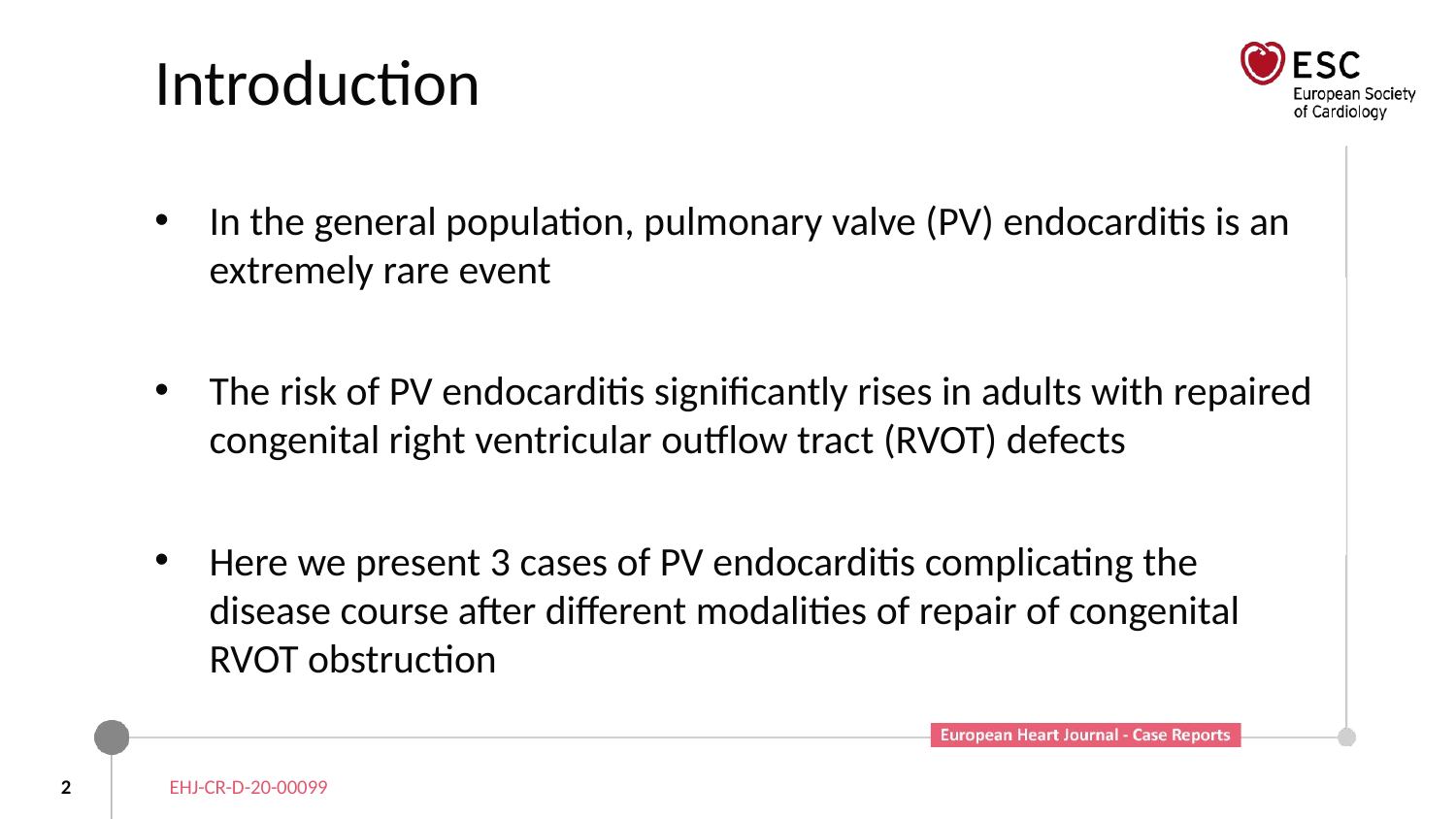

# Introduction
In the general population, pulmonary valve (PV) endocarditis is an extremely rare event
The risk of PV endocarditis significantly rises in adults with repaired congenital right ventricular outflow tract (RVOT) defects
Here we present 3 cases of PV endocarditis complicating the disease course after different modalities of repair of congenital RVOT obstruction
2
EHJ-CR-D-20-00099

## Slide 3
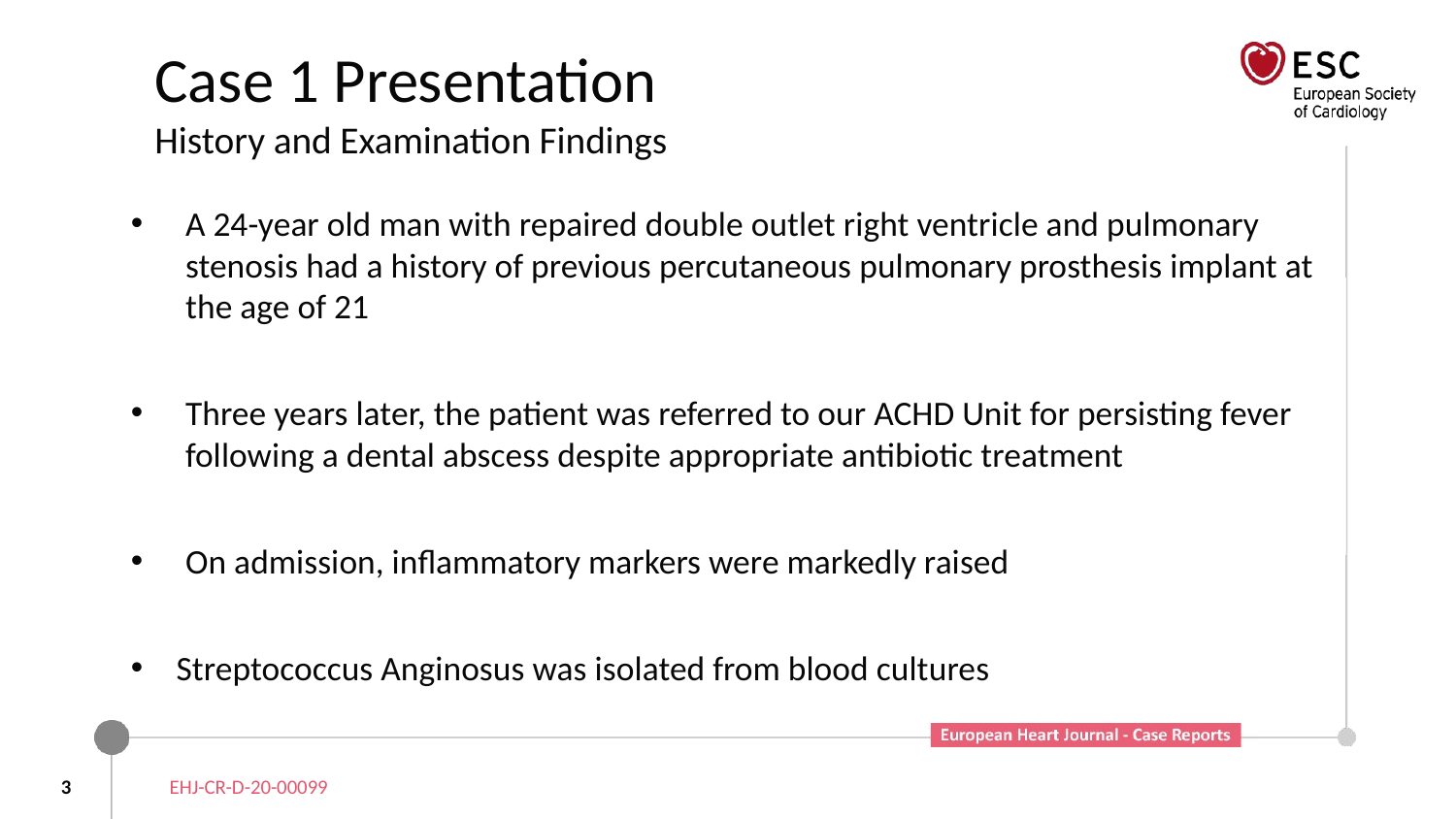

# Case 1 Presentation History and Examination Findings
A 24-year old man with repaired double outlet right ventricle and pulmonary stenosis had a history of previous percutaneous pulmonary prosthesis implant at the age of 21
Three years later, the patient was referred to our ACHD Unit for persisting fever following a dental abscess despite appropriate antibiotic treatment
On admission, inflammatory markers were markedly raised
Streptococcus Anginosus was isolated from blood cultures
3
EHJ-CR-D-20-00099

## Slide 4
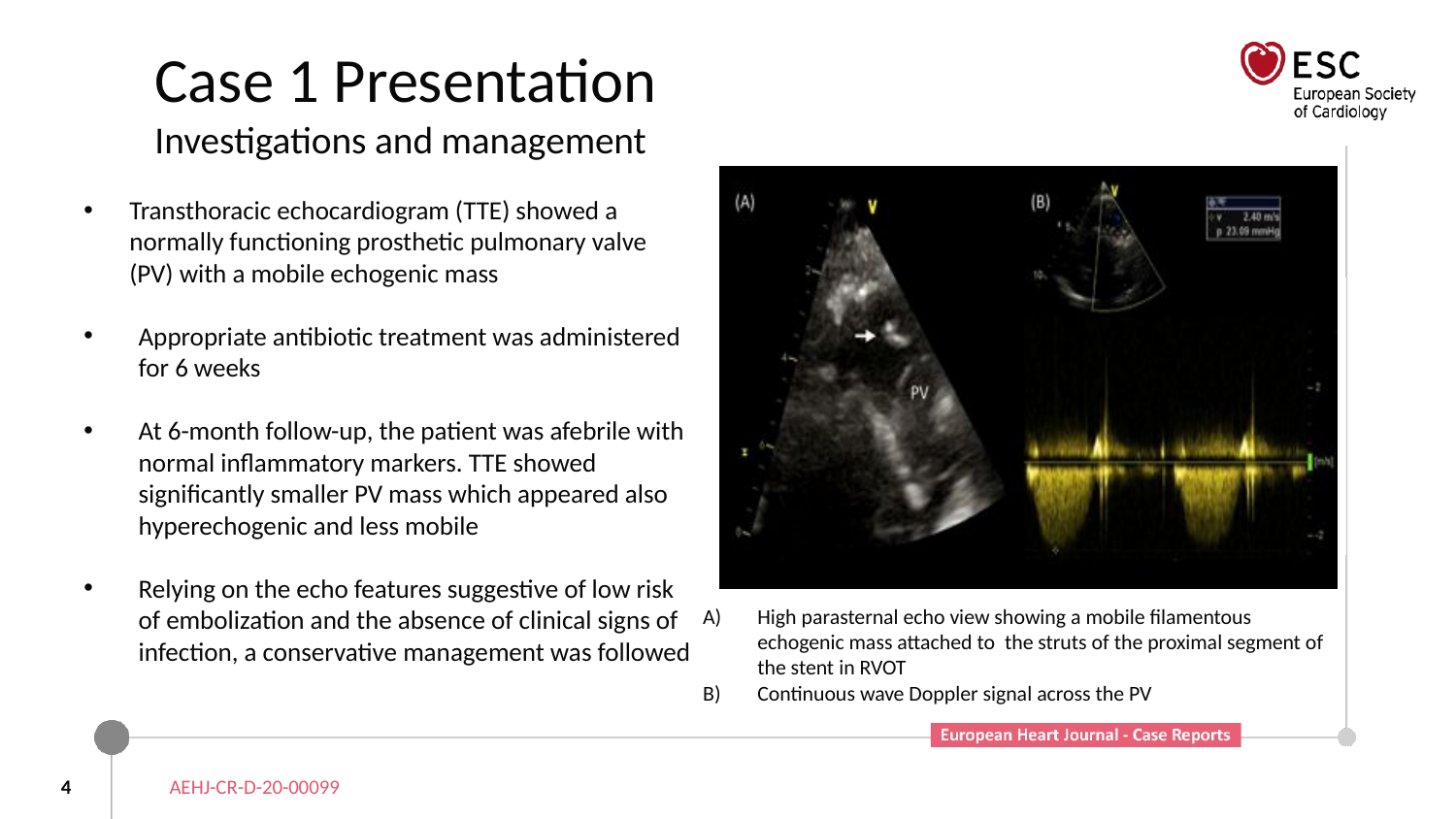

# Case 1 Presentation Investigations and management
Transthoracic echocardiogram (TTE) showed a normally functioning prosthetic pulmonary valve (PV) with a mobile echogenic mass
Appropriate antibiotic treatment was administered for 6 weeks
At 6-month follow-up, the patient was afebrile with normal inflammatory markers. TTE showed significantly smaller PV mass which appeared also hyperechogenic and less mobile
Relying on the echo features suggestive of low risk of embolization and the absence of clinical signs of infection, a conservative management was followed
High parasternal echo view showing a mobile filamentous echogenic mass attached to the struts of the proximal segment of the stent in RVOT
Continuous wave Doppler signal across the PV
4
AEHJ-CR-D-20-00099

## Slide 5
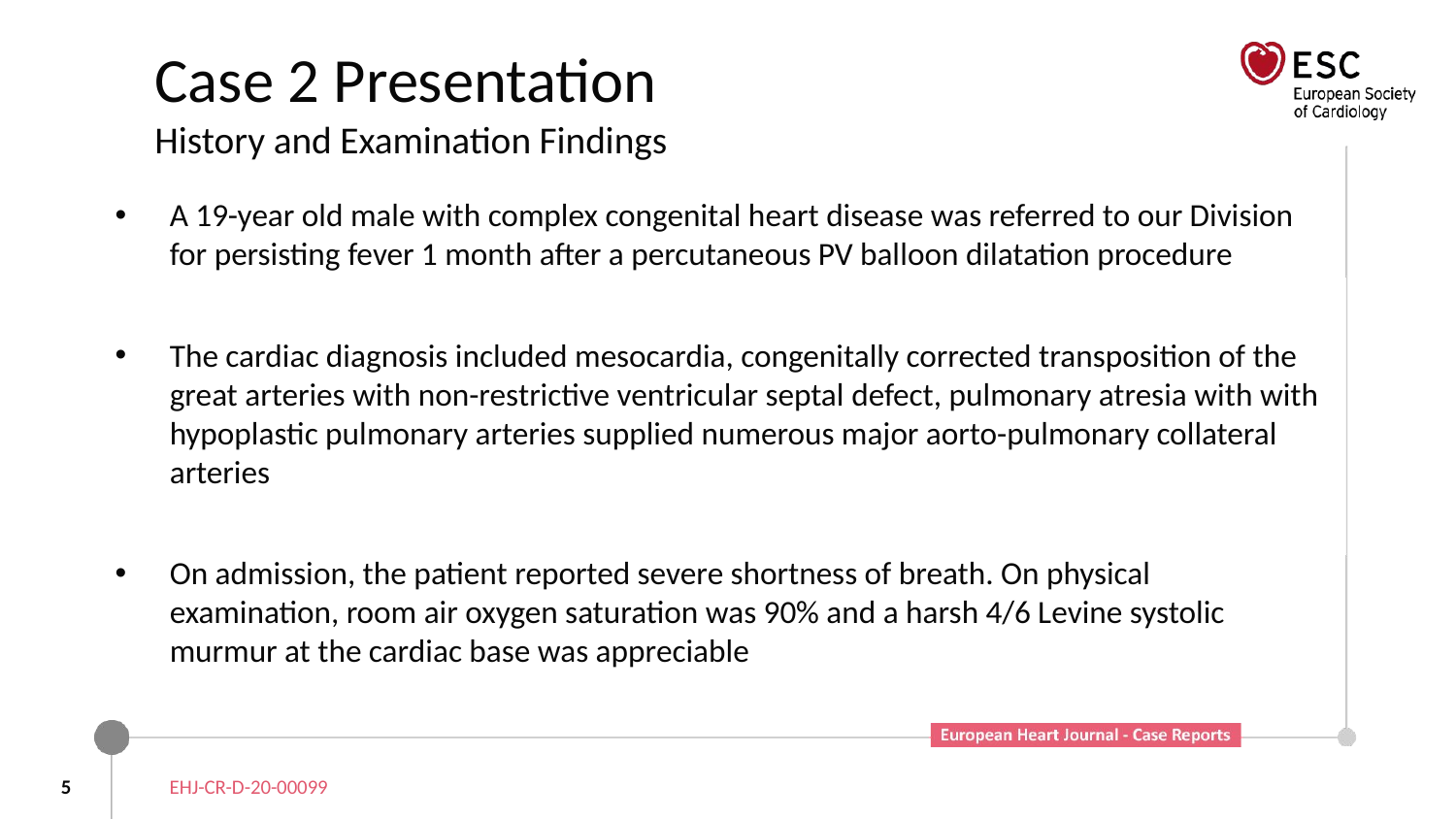

# Case 2 Presentation History and Examination Findings
A 19-year old male with complex congenital heart disease was referred to our Division for persisting fever 1 month after a percutaneous PV balloon dilatation procedure
The cardiac diagnosis included mesocardia, congenitally corrected transposition of the great arteries with non-restrictive ventricular septal defect, pulmonary atresia with with hypoplastic pulmonary arteries supplied numerous major aorto-pulmonary collateral arteries
On admission, the patient reported severe shortness of breath. On physical examination, room air oxygen saturation was 90% and a harsh 4/6 Levine systolic murmur at the cardiac base was appreciable
5
EHJ-CR-D-20-00099

## Slide 6
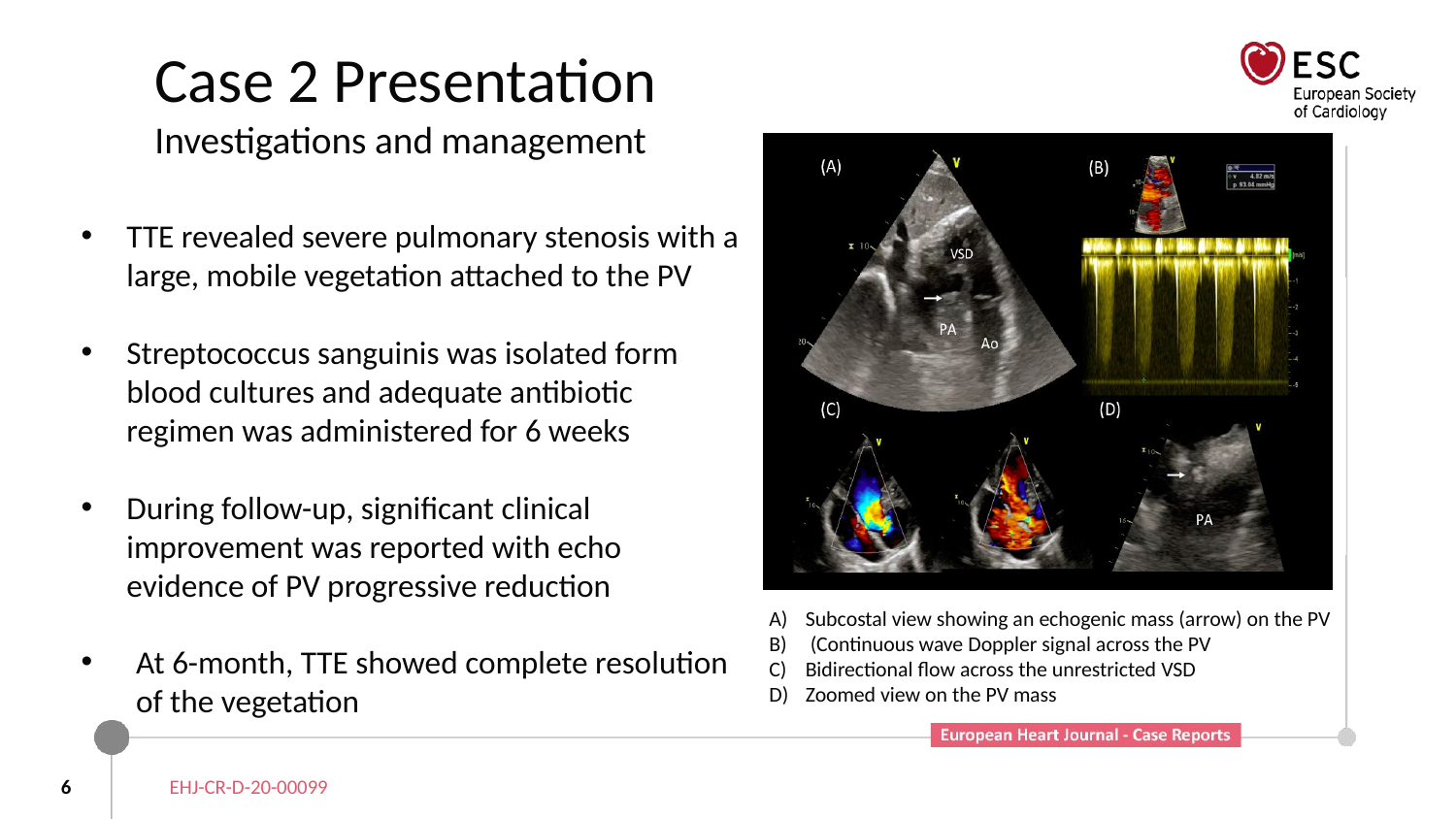

# Case 2 Presentation Investigations and management
TTE revealed severe pulmonary stenosis with a large, mobile vegetation attached to the PV
Streptococcus sanguinis was isolated form blood cultures and adequate antibiotic regimen was administered for 6 weeks
During follow-up, significant clinical improvement was reported with echo evidence of PV progressive reduction
At 6-month, TTE showed complete resolution of the vegetation
Subcostal view showing an echogenic mass (arrow) on the PV
 (Continuous wave Doppler signal across the PV
Bidirectional flow across the unrestricted VSD
Zoomed view on the PV mass
6
EHJ-CR-D-20-00099

## Slide 7
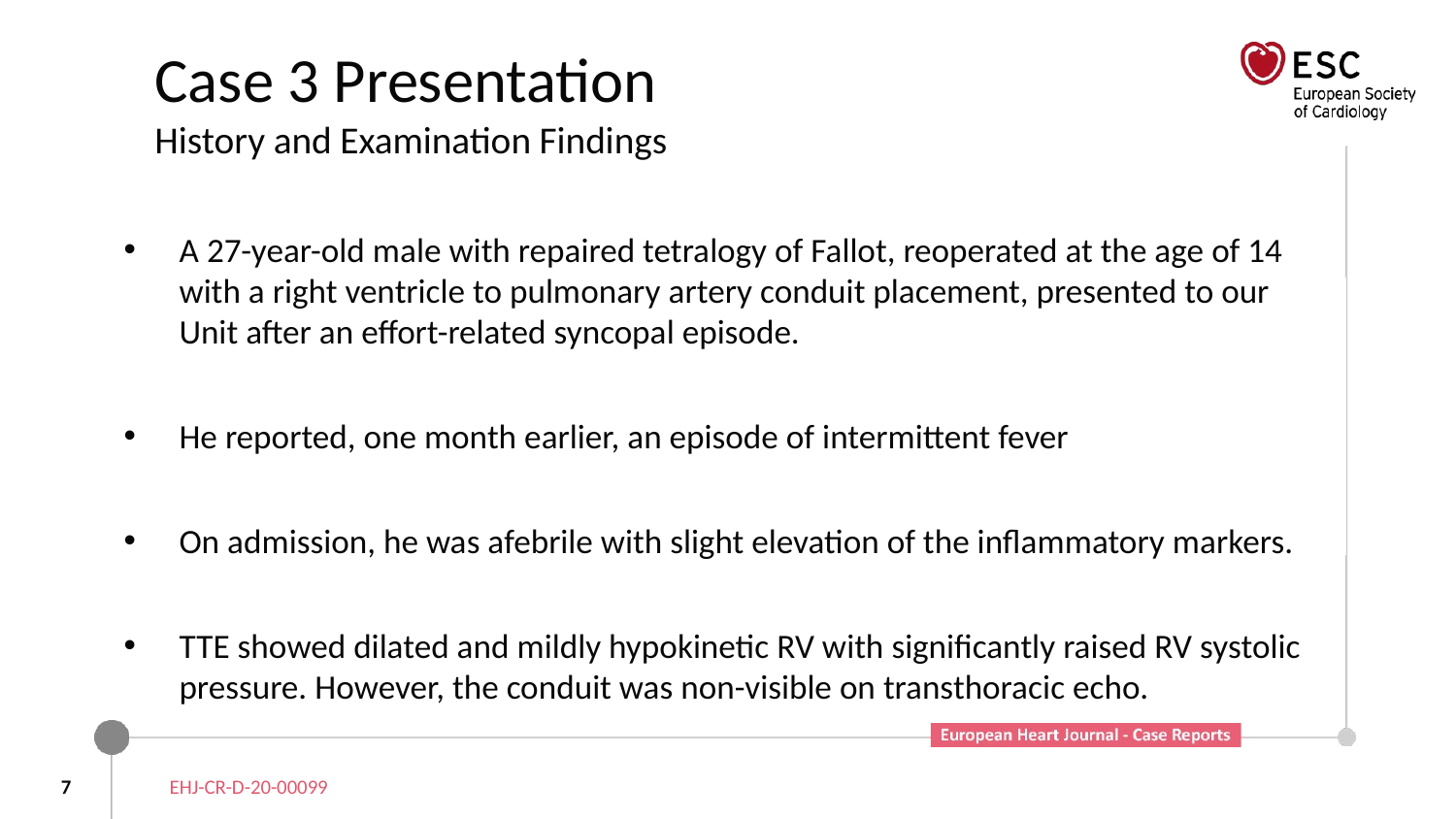

# Case 3 Presentation History and Examination Findings
A 27-year-old male with repaired tetralogy of Fallot, reoperated at the age of 14 with a right ventricle to pulmonary artery conduit placement, presented to our Unit after an effort-related syncopal episode.
He reported, one month earlier, an episode of intermittent fever
On admission, he was afebrile with slight elevation of the inflammatory markers.
TTE showed dilated and mildly hypokinetic RV with significantly raised RV systolic pressure. However, the conduit was non-visible on transthoracic echo.
7
EHJ-CR-D-20-00099

## Slide 8
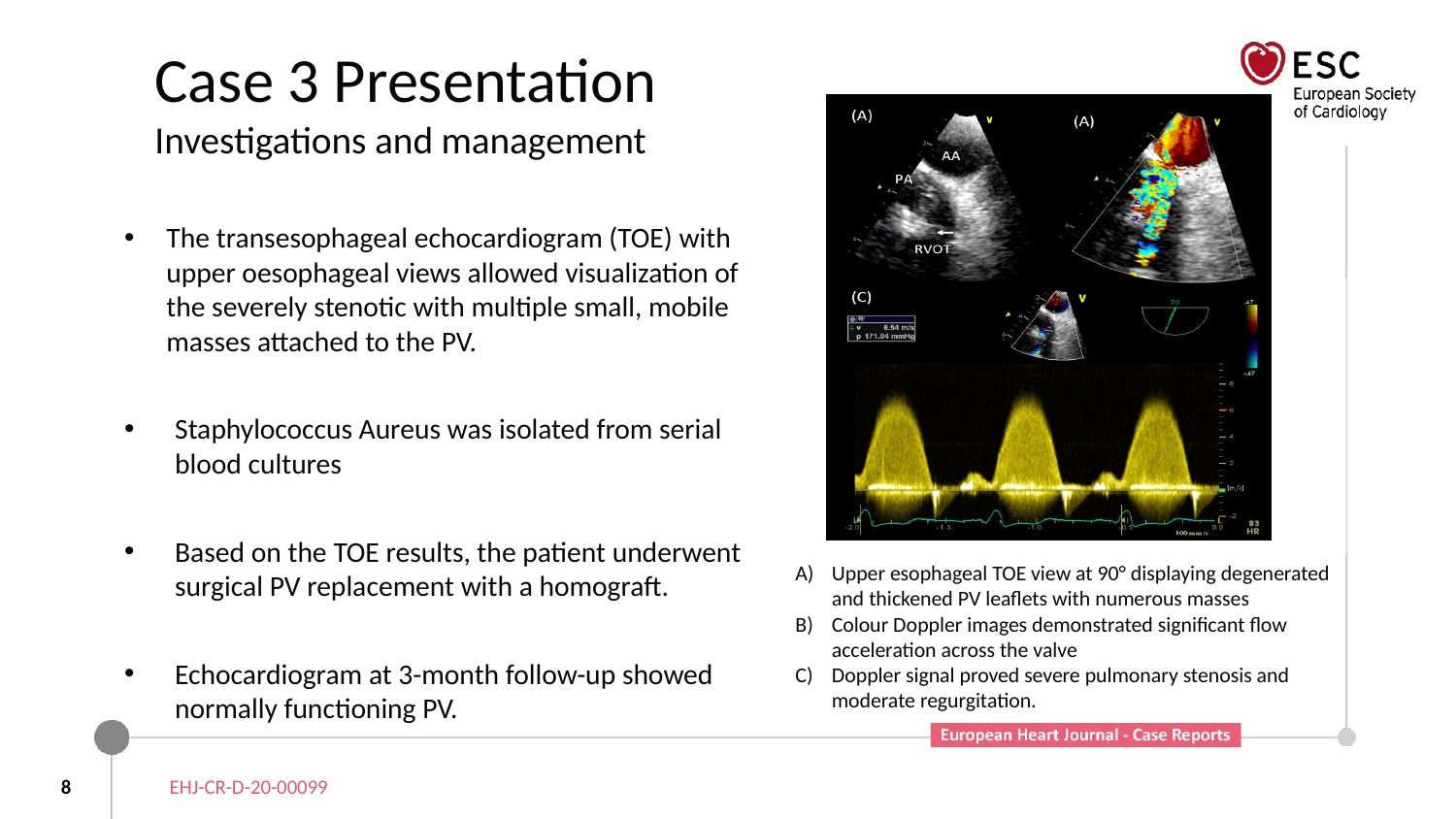

# Case 3 Presentation Investigations and management
The transesophageal echocardiogram (TOE) with upper oesophageal views allowed visualization of the severely stenotic with multiple small, mobile masses attached to the PV.
Staphylococcus Aureus was isolated from serial blood cultures
Based on the TOE results, the patient underwent surgical PV replacement with a homograft.
Echocardiogram at 3-month follow-up showed normally functioning PV.
Upper esophageal TOE view at 90° displaying degenerated and thickened PV leaflets with numerous masses
Colour Doppler images demonstrated significant flow acceleration across the valve
Doppler signal proved severe pulmonary stenosis and moderate regurgitation.
8
EHJ-CR-D-20-00099

## Slide 9
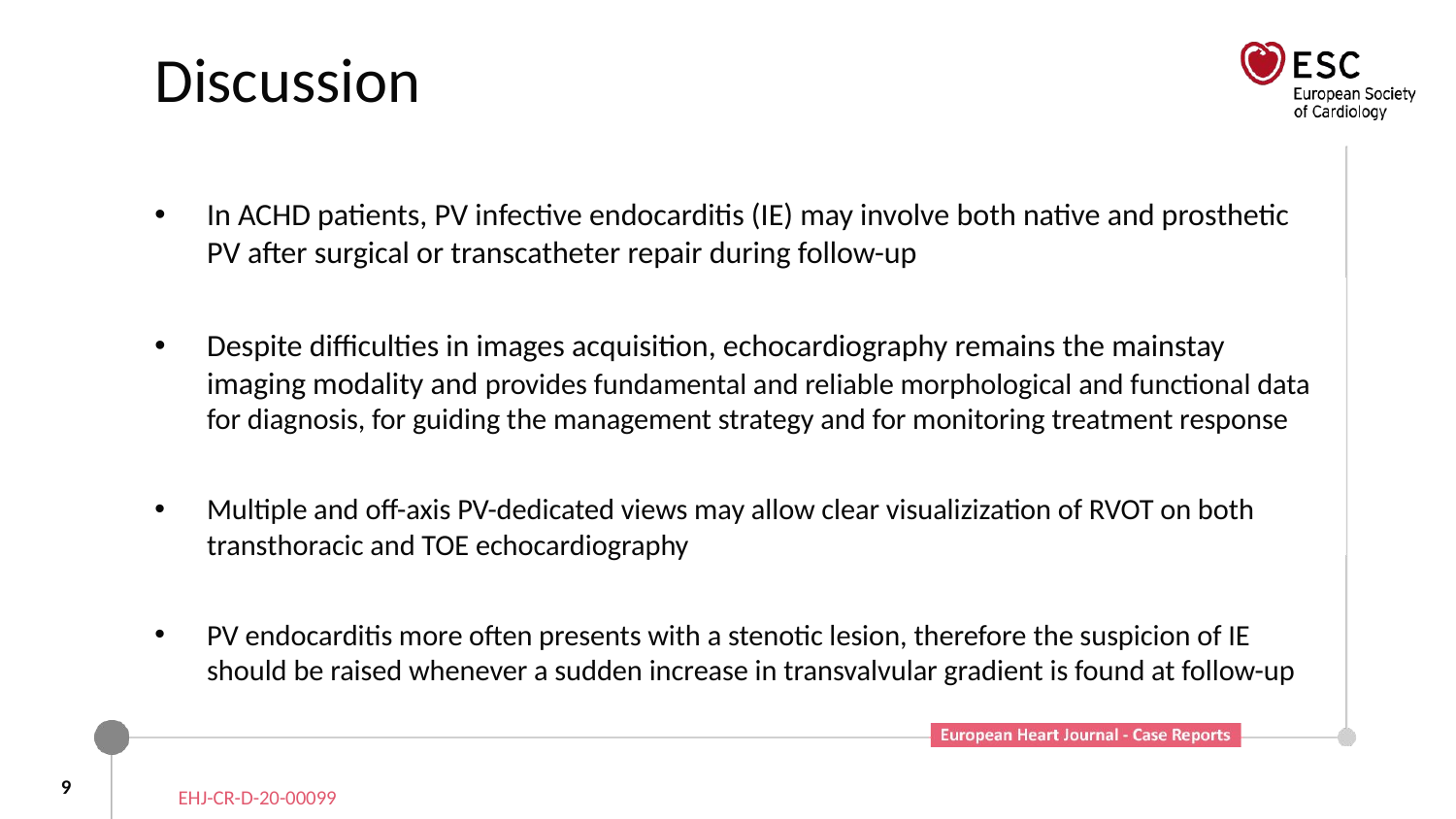

# Discussion
In ACHD patients, PV infective endocarditis (IE) may involve both native and prosthetic PV after surgical or transcatheter repair during follow-up
Despite difficulties in images acquisition, echocardiography remains the mainstay imaging modality and provides fundamental and reliable morphological and functional data for diagnosis, for guiding the management strategy and for monitoring treatment response
Multiple and off-axis PV-dedicated views may allow clear visualizization of RVOT on both transthoracic and TOE echocardiography
PV endocarditis more often presents with a stenotic lesion, therefore the suspicion of IE should be raised whenever a sudden increase in transvalvular gradient is found at follow-up
9
EHJ-CR-D-20-00099

## Slide 10
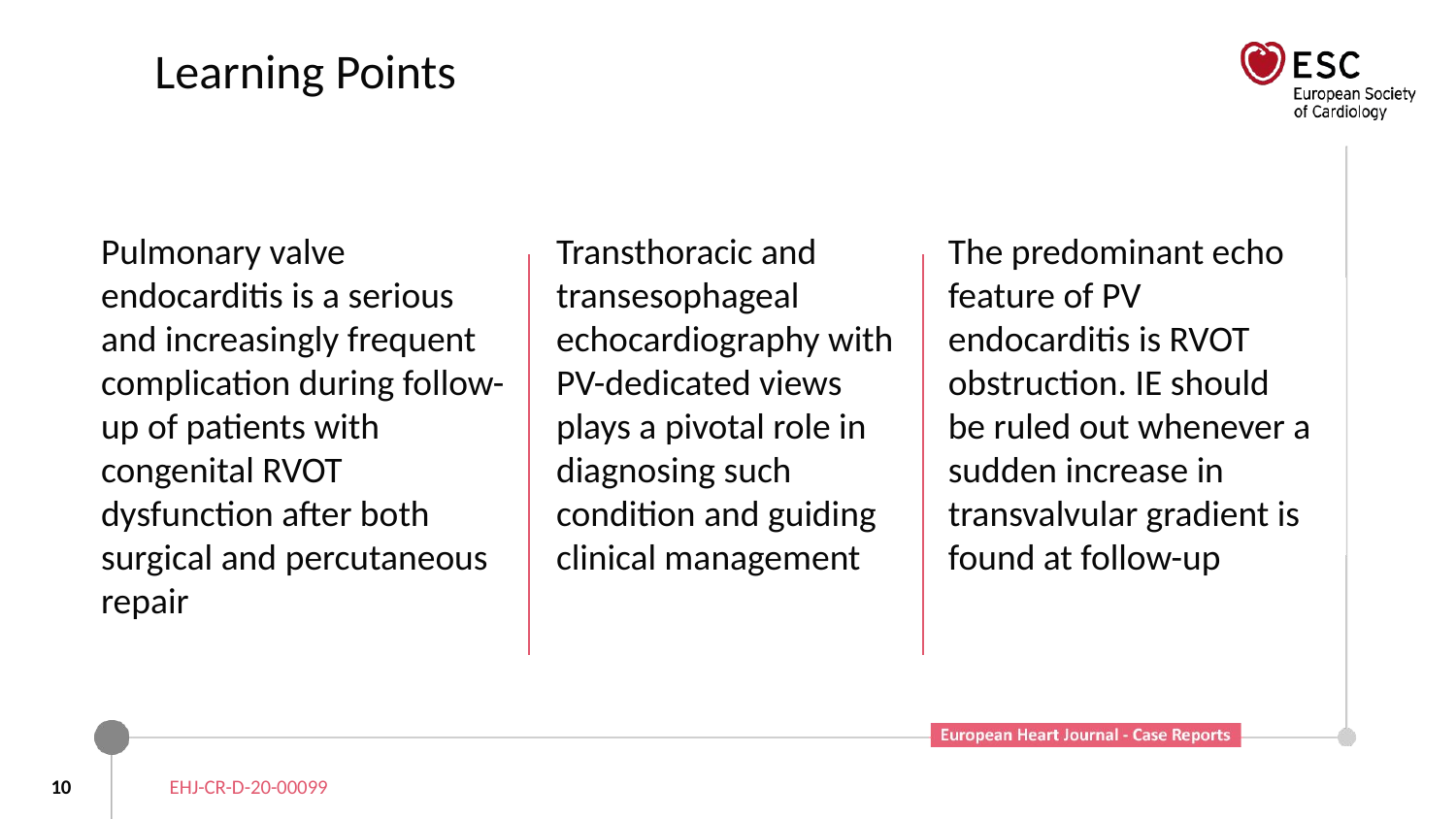

# Learning Points
The predominant echo feature of PV endocarditis is RVOT obstruction. IE should be ruled out whenever a sudden increase in transvalvular gradient is found at follow-up
Transthoracic and transesophageal echocardiography with PV-dedicated views plays a pivotal role in diagnosing such condition and guiding clinical management
Pulmonary valve endocarditis is a serious and increasingly frequent complication during follow-up of patients with congenital RVOT dysfunction after both surgical and percutaneous repair
10
EHJ-CR-D-20-00099

## Slide 11
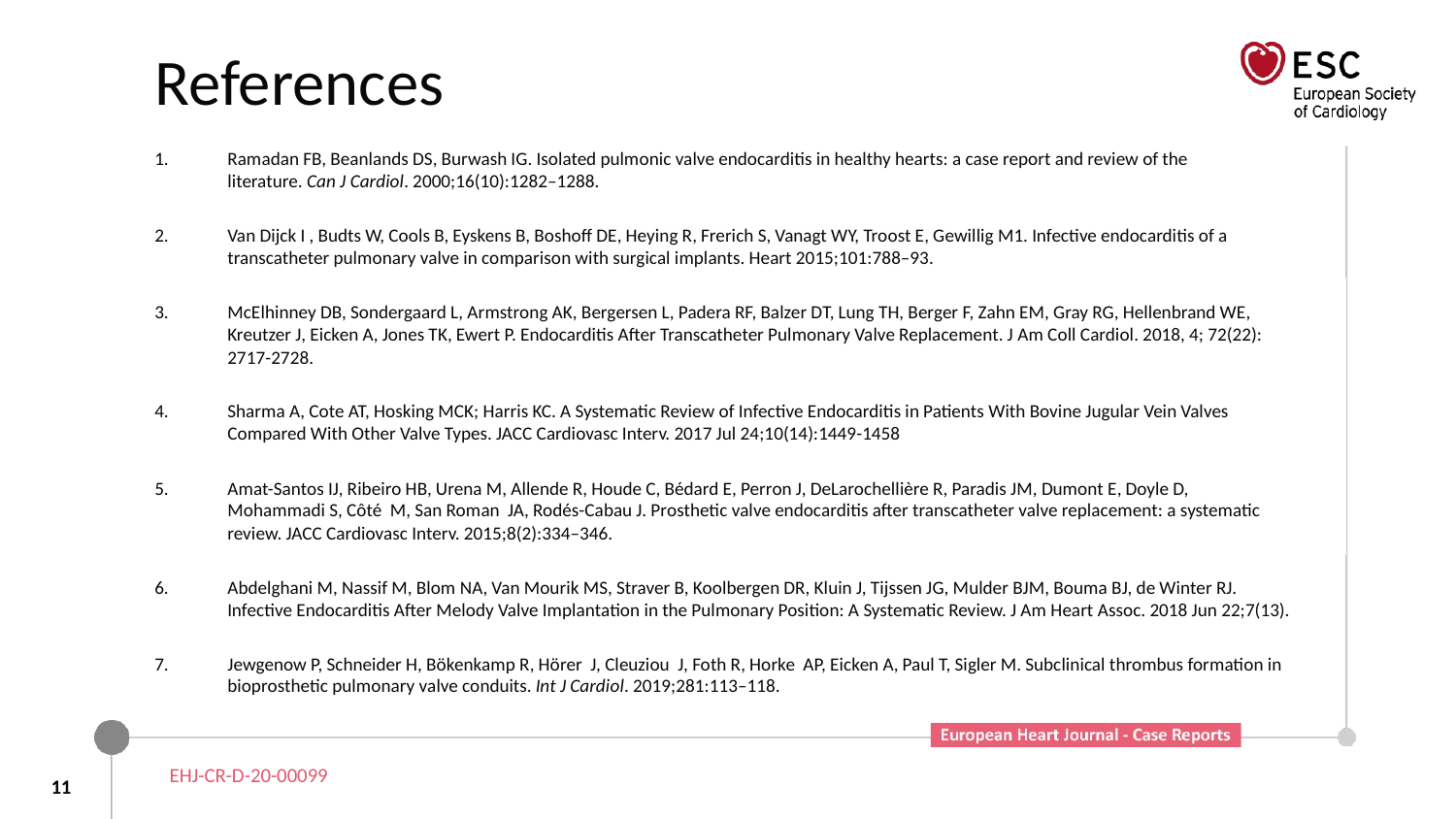

# References
Ramadan FB, Beanlands DS, Burwash IG. Isolated pulmonic valve endocarditis in healthy hearts: a case report and review of the literature. Can J Cardiol. 2000;16(10):1282–1288.
Van Dijck I , Budts W, Cools B, Eyskens B, Boshoff DE, Heying R, Frerich S, Vanagt WY, Troost E, Gewillig M1. Infective endocarditis of a transcatheter pulmonary valve in comparison with surgical implants. Heart 2015;101:788–93.
McElhinney DB, Sondergaard L, Armstrong AK, Bergersen L, Padera RF, Balzer DT, Lung TH, Berger F, Zahn EM, Gray RG, Hellenbrand WE, Kreutzer J, Eicken A, Jones TK, Ewert P. Endocarditis After Transcatheter Pulmonary Valve Replacement. J Am Coll Cardiol. 2018, 4; 72(22): 2717-2728.
Sharma A, Cote AT, Hosking MCK; Harris KC. A Systematic Review of Infective Endocarditis in Patients With Bovine Jugular Vein Valves Compared With Other Valve Types. JACC Cardiovasc Interv. 2017 Jul 24;10(14):1449-1458
Amat-Santos IJ, Ribeiro HB, Urena M, Allende R, Houde C, Bédard E, Perron J, DeLarochellière R, Paradis JM, Dumont E, Doyle D, Mohammadi S, Côté M, San Roman JA, Rodés-Cabau J. Prosthetic valve endocarditis after transcatheter valve replacement: a systematic review. JACC Cardiovasc Interv. 2015;8(2):334–346.
Abdelghani M, Nassif M, Blom NA, Van Mourik MS, Straver B, Koolbergen DR, Kluin J, Tijssen JG, Mulder BJM, Bouma BJ, de Winter RJ. Infective Endocarditis After Melody Valve Implantation in the Pulmonary Position: A Systematic Review. J Am Heart Assoc. 2018 Jun 22;7(13).
Jewgenow P, Schneider H, Bökenkamp R, Hörer J, Cleuziou J, Foth R, Horke AP, Eicken A, Paul T, Sigler M. Subclinical thrombus formation in bioprosthetic pulmonary valve conduits. Int J Cardiol. 2019;281:113–118.
11
EHJ-CR-D-20-00099
